# The impact of thermal effects on filamentation and supercontinuum generation in bulk materials at MHz pulse repetition rates

**DOI:** 10.1038/s41598-026-51931-y

**Published:** 2026-05-12

**Authors:** Vytautas Jukna, Matas Šutovas, Vaida Marčiulionytė, Gintaras Tamošauskas, Audrius Dubietis

**Affiliations:** Laser Research Center, Vilnius University, Saulėtekio Avenue 10, 10223 Vilnius, Lithuania

**Keywords:** Optics and photonics, Physics

## Abstract

We experimentally study the impact of beam focusing geometry-related heat accumulation on supercontinuum spectra in YAG crystal pumped with 210 fs, 1030 nm pulses by varying pulse repetition rate in the 200 kHz - 2 MHz range. It is demonstrated that in the case of converging pump beam, the red-shifted (long-wavelength) portion of SC spectrum shrinks considerably with the increase of pulse repetition rate, almost completely vanishing at 2 MHz repetition rate, and such spectral shrinking takes place on a very fast, microsecond time-scale. In contrast, in the case of diverging pump beam, stable and broad supercontinuum spectrum is generated regardless of pulse repetition rate. The contrasted supercontinuum generation performances are attributed to large differences of deposited energy due to nonlinear losses experienced by converging and diverging pump beams, which set different thermal regimes of beam filamentation. The experimental observations were backed-up with numerical simulations unveiling the role of heat accumulation and thermal lensing on the nonlinear propagation of the leading pulse, which is responsible for red-shifted spectral broadening. Our findings are of importance for better understanding of underlying phenomena and optimization of supercontinuum generation setups driven by the state-of-the-art high repetition rate ultrafast Yb lasers.

## Introduction

The generation of supercontinuum (SC) in bulk materials represents an experimentally simple and robust technique for the generation of coherent radiation with an octave-spanning frequency spectrum; see, e.g.^1^. By its nature, SC generation is a highly dynamic nonlinear process, driven by collective action of self-focusing, self-phase-modulation, self-steepening, multiphoton absorption and generation of free electron plasma, which govern nonlinear propagation of an ultrashort laser pulse in the entire space-time domain. The achievable extent of spectral broadening is important for practical applications and generally depends on the physical properties of nonlinear material, such as energy bandgap, dispersion, and nonlinearity^2–4^. In addition to material properties, the overall performance of SC generation could be optimized by experimental settings, such as the wavelength, duration, and energy of the driving pulses, the length of the nonlinear medium, and ultimately the focusing geometry of the pump beam^5–8^. The latter is of importance for the general dynamics of spectral broadening^9,10^ and offers an additional degree of freedom to optimize the relevant properties of generated broadband radiation. More specifically, loose focusing of the pump beam yields enhanced red-shifted broadening of the SC spectrum^11,12^, while using a converging or diverging pump beam (i.e., placing the nonlinear material before or after focal plane of the focusing lens) allows to optimize either blue-shifted or red-shifted extent of SC spectrum, respectively^13,14^. Moreover, SC generation with diverging pump beams enables suppression of multiple filamentation and laser-induced damage in the case of high pump pulse energies used^15^. The divergence of the input pump beam has an impact on the coherence properties of SC radiation^14^, as well as the stability of the spectral phase^16^ and the wavelength jitter^17^, which are crucial parameters for practical applications, such as providing high quality seed signal for optical parametric amplifiers operating in a few optical cycle regime.

However, SC generation in bulk materials at high pulse repetition rates (100s of kHz to a few MHz) imposes a number of fundamental and practical issues, which arise from the very nature of femtosecond filamentation process, where a certain fraction of pulse energy is deposited to the transparent material via multiphoton absorption, free carrier absorption and impact ionization, see^18,19^. This in turn promotes the formation of transient defect states, such as self-trapped excitons (STEs) and color centers^20^, as well as induces local and bulk heating^21^. If the decay times of defect states are longer than the time interval between adjacent pulses, defects accumulate with the passage of every subsequent laser pulse, evolving into permanent material modifications via local lattice rearrangement, resulting in refractive index change^22^ and formation of self-organized laser-induced periodic structures (nanogratings) in volume^23,24^. This material modification alters the nonlinear propagation of the laser pulse, which is manifested by gradual shrinking and extinction of the SC spectrum, and ultimately, termination of filamentation process^25^. Although permanent material modification is not induced, thermal effects alone play an important role in the nonlinear propagation dynamics, affecting relevant properties of SC radiation. In that regard, recent studies of high repetition rate filamentation in air revealed that the increased effect of thermal coupling between successive pulses leads to a reduction in the breakdown potential^26^, a reduction in air density, and a change in refractive index caused by localized heat deposition^27^. A reduced air density within the filamenting region was shown to increase the peak intensity in the filament and its length, and consequently to produce more efficient third harmonic generation and nitrogen fluorescence^28^. Reduction of the critical power for self-focusing^29^ and enhanced spectral blue shift of the SC emission^30^ with an increase of pulse repetition rate were also observed. For filamentation in condensed media, thermally-induced instabilities of the SC spectrum were studied during SC generation in water and suppressed by using a constant laminar flow of the liquid^31^, while partial mitigation of thermally-induced decay of SC radiation in bulk solid-state material (sapphire) was demonstrated by temperature control of the nonlinear crystal^32^. The observation of thermal lensing was reported at sub-MHz pulse repetition rates during filamentation in diamond^33^. Local heating within filamentary regions in synthetic silica glass was studied in relation to material processing applications^34^. However, the reported results present only a very fragmented picture of heat-induced effects in femtosecond filamentation, and their role in practical schemes for SC generation in bulk solids at MHz pulse repetition rates^35–37^ has not received adequate attention so far.

In the present Paper, we conducted an experimental and numerical study addressing the impact of thermal effects on spectral features of bulk-generated SC using high repetition rate femtosecond Yb-laser source. The experiments were performed in a YAG crystal, which stands out as an excellent nonlinear material, widely employed for SC generation with pump pulse durations ranging from a few optical cycles to a few picoseconds, and demonstrated robust performance with a variety of driving wavelengths provided by the state-of-the-art ultrafast laser sources, see e.g.^11,13,35,38–45^. Our study demonstrates significant differences in SC spectral extent and uncovers contrasting time evolutions of SC spectra due to heat accumulation with converging and diverging pump beams at sub-MHz and MHz pulse repetition rates.

## Experimental setup

**Fig. 1:**
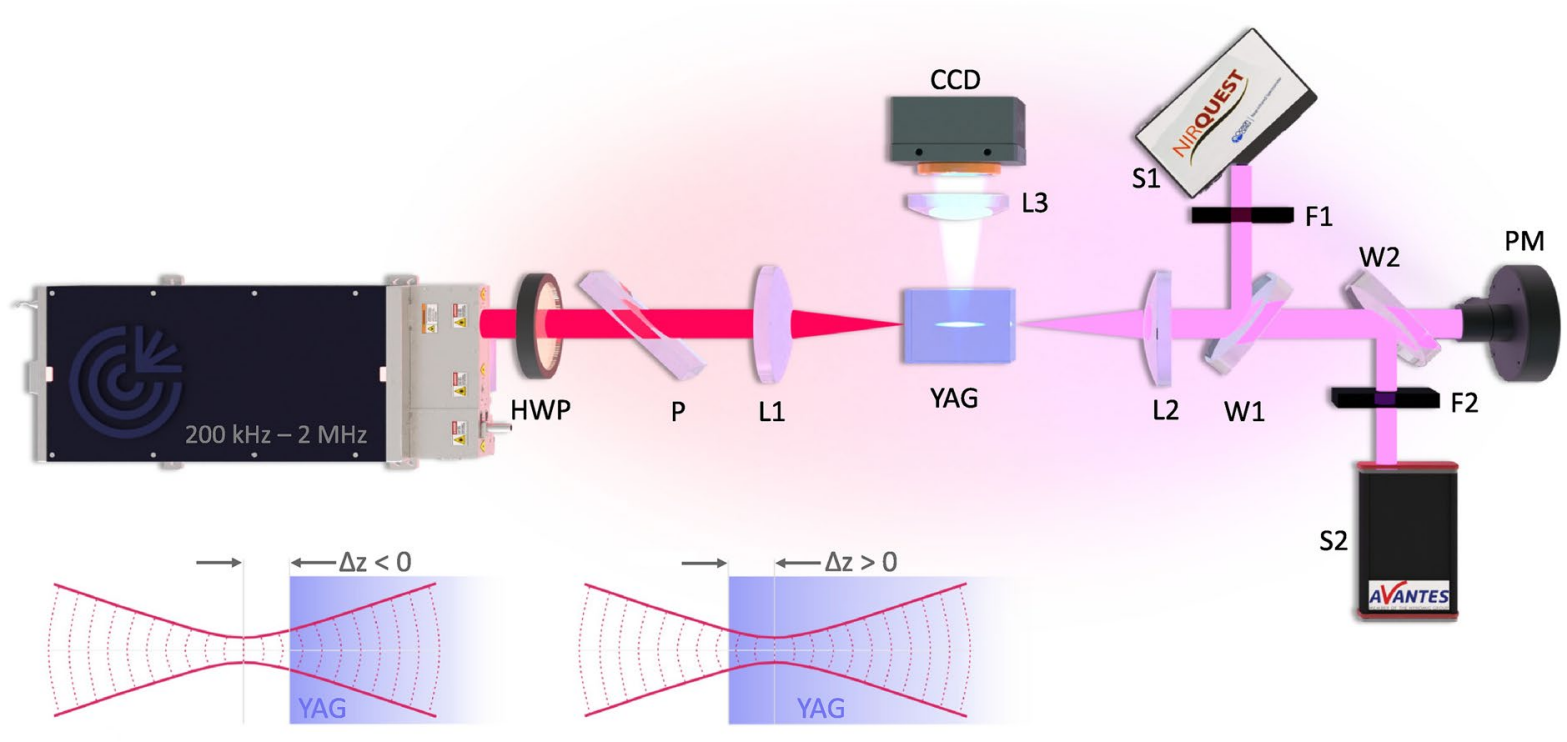
Experimental setup: HWP, half-wave plate; P, polarizer; L1, focusing lens; L2, collimating lens; L3, imaging lens; W1, W2, fused silica wedges; F1, long-pass filter; F2, short-pass filter; SP1, NIR-SWIR spectrometer; SP2, UV-VIS-NIR spectrometer; CCD, CCD camera; PM, power meter. The inset schematically illustrates the positions of beam waist with respect to the entrance face of sample for diverging ($\Delta z<0$) and converging ($\Delta z>0$) pump beams.

The laser source was an amplified Yb:KGW laser (Carbide, Light Conversion), which delivered 210 fs pulses with a central wavelength of 1030 nm. The experimental setup is sketched in Fig. 1. The linearly polarized pump beam with 4.1 mm diameter (at the $1/e^2$ intensity level) was focused with a lens L1 ($f=+125$ mm) to an undoped and uncoated YAG sample of 10 mm thickness. The focusing lens was mounted on a translation stage for fine tuning of the position of its geometrical focus with respect to the entrance face of the crystal, as depicted in the inset. After suitable attenuation by means of a half-wave plate (HWP) and polarizer (P), the pump pulse energy of $1.28~\mu$J (measured before the focusing lens) was set to induce a single filament and SC generation over a wide range of lens positions and kept constant during all measurements, where the pulse repetition rate varied from 200 kHz to 2 MHz.

The lens L2 was used to recollimate the output radiation. SC spectra were measured with NIR-SWIR (NIRQuest-512, Ocean Optics) and UV-VIS-NIR (AvaSpec-3648, Avantes) spectrometers (SP1 and SP2), with respective detection ranges of $0.2-1.1~\upmu$m and $0.9-2.1~\upmu$m, taking reflections from fused silica wedges W1, W2 and appropriate long-pass (F1) and short-pass (F2) filters for recording the short-wavelength and long-wavelength portions of SC, respectively. The full SC spectra were reconstructed after correction for filter transmission and spectrometer detector sensitivity functions. The nonlinear losses were evaluated from the measurements of transmitted energy, by collecting the output radiation onto a thermopile power meter PM (Ophir) and taking into account Fresnel reflection losses. The shape of the filament and the position of the nonlinear focus were monitored by broadband luminescence of the YAG crystal, which peaks at around 300 nm and is attributed to a combination of self-trapped exciton and antisite defect-related emissions^46^. The filament-induced luminescence traces were imaged with a lens L3 through the polished side of the crystal and recorded with a CCD camera (Point Grey GS2-GE-20S4M-C).

The pulse-to-pulse spectral dynamics at the long-wavelength side of the SC spectrum was recorded with a fast InGaAs photodiode (MTPD1346D-150, Marktech) connected to an oscilloscope (not shown). In these measurements, the spectral window of $1500-1700$ nm was set by a combination of a band-pass filter (FB1750-500, Thorlabs), which had a transmission range of 500 nm centered at 1750 nm, and a detection range of a photodiode, which had a long-wavelength cut-off at 1700 nm.

## Results and discussion

### Filamentation and SC generation with converging and diverging pump beams

**Fig. 2:**
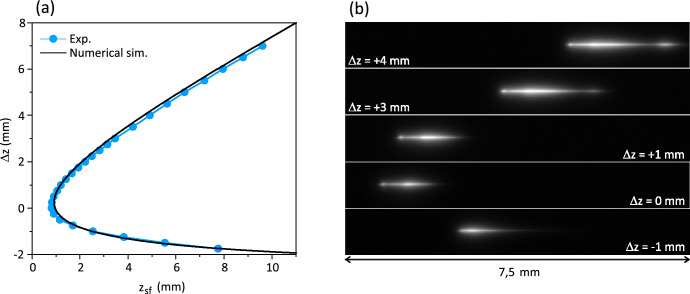
(**a**) Position of the nonlinear focus, $z_\textrm{sf}$, as a function of the pump beam waist offset with respect to the entrance face of the crystal $\Delta z$. Circles show experimental data, solid curve shows the best fit obtained with Marburger‘s formula and taking into account the curvature of the input beam phase front. (**b**) Examples of measured filament-induced luminescence traces for different offset values.

To start with, we first explored the relevant features of filamentation and SC generation with a fixed pump pulse energy of $1.28~\upmu$J and pulse repetition rate of 200 kHz. Figure 2a shows the experimentally measured position of nonlinear focus ($z_\textrm{sf}$) as a function of the offset of the waist of the pump beam with respect to the entrance face of the crystal ($\Delta z$). Here, the waist position of the pump beam is defined in air, as schematically illustrated in the inset of Fig. 1. The zero offset ($\Delta z=0$) corresponds to a position of the beam waist that is exactly located on the entrance face of the sample. The negative offset values denote the position of the beam waist before the entrance face of the sample, while the positive values denote the position of the beam waist, which is located inside the crystal, corresponding to the conditions of entering the nonlinear material with diverging and converging pump beams, respectively. The position of the nonlinear focus was defined as a first intensity maximum of filament-induced luminescence traces, whose typical examples are shown in Fig. 2b. The solid curve shows the best fit to the experimental data using Eq. (4), where the value of $n_2$ was taken as a free parameter. It is worth noticing that the shape and length of filament luminescence traces change considerably with the offset position of the pump beam waist. An apparently shorter and smoother filament was produced with the diverging pump beam ($\Delta z=-1$ mm), whereas the converging pump beam produced a visually longer filament with a more pronounced intensity variation along the propagation path. Filament refocusing was observed when the pump beam was focused deep inside the crystal, e.g. at $\Delta z=+3$ mm and $\Delta z=+4$ mm, while no filament was formed and no SC was generated for $\Delta z<-2$ mm and $\Delta z>+7.5$ mm, as the nonlinear focus shifted outside the sample.

**Fig. 3:**
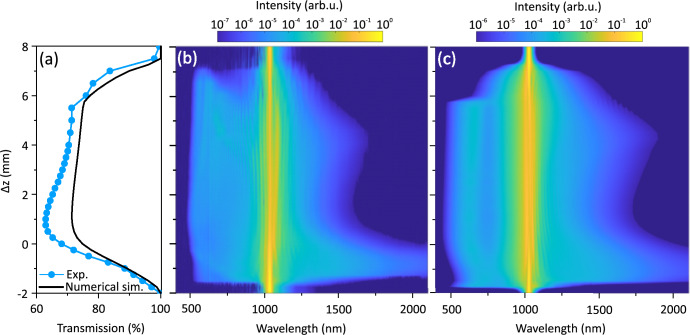
(**a**) Experimentally measured (circles) and numerically simulated (solid curve) transmission of YAG sample as a function of beam waist offset and corresponding (**b**) measured, (**c**) numerically simulated dynamics of spectral broadening.

Figure 3a compares the measured and calculated transmissions of the YAG crystal as functions of the offset of the beam waist. The decrease in transmittance is associated with the onset of nonlinear losses, which are a distinctive feature of beam filamentation process and originate from six photon absorption (assuming pump photon energy of 1.2 eV and YAG bandgap of 6.5 eV) and free electron plasma absorption via inverse Bremsstrahlung effect. Despite a slight difference between the experimentally measured and numerically simulated transmission values, both transmission curves exhibit an essentially identical character. The transmission data are linked to the SC spectra measured experimentally (Fig. 3b) and simulated numerically (Fig. 3c), showing a good agreement between the experimental and numerical data for the entire range of offset values. The SC spectra with the largest red-shifts are generated when entering the nonlinear material with diverging pump beam, in a relatively narrow offset value range from -1.5 to -0.5 mm, which aligns with the descending slope of transmission curve. The nonlinear losses increase rapidly toward the zero offset (where the position of geometrical focus is located on the front face of the sample) and are the largest at around $\Delta z=+1$ mm, where the SC spectra with the narrowest red-shifted broadenings are generated. Interestingly, in this offset range, the SC spectra exhibit slightly larger blue-shifted broadenings. With focusing the pump beam deeper inside the sample, the nonlinear losses start slowly decreasing, but still remaining at sufficiently high level. The red-shifted spectral broadening slightly recovers at around $\Delta z=+4$ mm, however, the SC spectrum develops modulation, which is best pronounced at the short-wavelength side (this feature was not captured in the simulation). The spectral modulation is attributed to filament refocusing, as confirmed by filament-induced luminescence traces measured at $\Delta z=+3$ mm and $\Delta z=+4$ mm, see Fig. 2b.

**Fig. 4:**
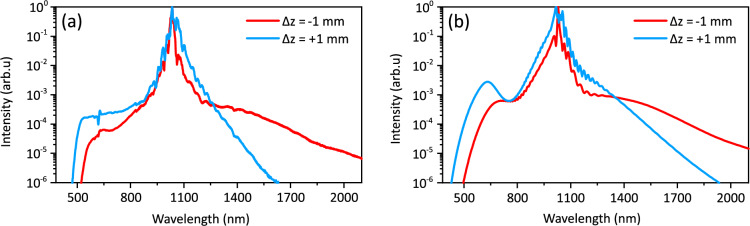
(**a**) Experimentally measured and (**b**) numerically simulated supercontinuum spectra produced with converging ($\Delta z=+1$ mm) and diverging ($\Delta z=-1$ mm) pump beams.

These results summarize in detail the earlier findings presented elsewhere^13,14^, also demonstrating how sensitive is the spectral extent of generated SC to the pump beam waist position with respect to the entrance face of the nonlinear material, especially bearing in mind that the Rayleigh length of the pump beam is 1.22 mm. Figure 4 compares characteristic SC spectra generated with converging and diverging pump beams. The offset values $\Delta z=+1$ mm and $\Delta z=-1$ mm were chosen as representative, where SC spectra with the largest either blue or red spectral shifts were produced, respectively.

**Fig. 5:**
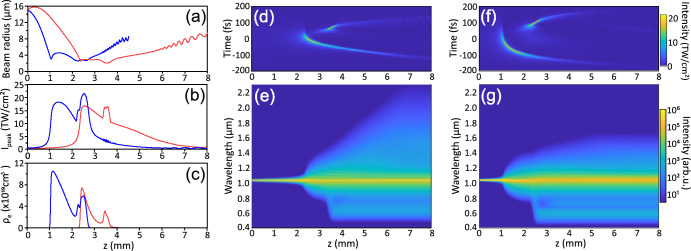
The dynamics of (**a**) beam radius, (**b**) peak intensity and (**c**) plasma density at the beam center versus propagation distance *z* for diverging ($\Delta z=-1$ mm, red curves) and converging ($\Delta z=+1$ mm, blue curves) pump beams. (**d**,**e**) The temporal and spectral evolutions of diverging pump beam, respectively, while (**f,g**) show the corresponding evolutions of converging pump beam.

In order to get a deeper insight into these findings, we refer to numerical simulations, where we compare two distinctive cases of spectral broadening in more detail. Figure 5 presents the relevant dynamics of the HWHM beam radius, peak intensity, and plasma density at the center of the beam, as well as the corresponding temporal and spectral evolutions of diverging ($\Delta z=-1$ mm) and converging ($\Delta z=+1$ mm) pump beams versus the propagation distance *z*. First of all, we note that self-focusing and filamentation of relatively long (200 fs) input pulse undergoes rather specific temporal and spectral dynamics, which are described in more detail elsewhere^10^. More specifically, the self-focusing of this pulsed beam produces two well-defined nonlinear foci (Fig. 5a) with corresponding intensity (Fig. 5b) and plasma density (Fig. 5c) peaks. The distinctive feature of nonlinear propagation of the input pulse is its spatio-temporal reshaping that starts at the vicinity of the nonlinear focus. The free electron plasma produced by the leading part of the pulse absorbs and defocuses its rear part, so the intensity peak of the pulse shifts toward the front of the time frame. At the center of the beam, the pulse (which in what follows will be referred to as the leading pulse) experiences plasma-induced self-shortening, which with propagation produces gradual red-shifted spectral broadening in the spectral domain, see e.g. Fig. 5d, e. With further propagation, the peak intensity of the leading pulse decreases, the plasma density reduces, and the effect of plasma defocusing ceases. Shortly after, the trailing pulse at the beam center is replenished, which then self-focuses producing secondary intensity and plasma peaks. To be precise, at the secondary nonlinear focus, the replenished pulse undergoes an asymmetric splitting that produces an intense trailing sub-pulse (that is shifted to the back of the time frame) which is responsible for explosive blue-shifted spectral broadening and a weak leading sub-pulse, whose effect on the red-shifted spectral broadening is very minor. Therefore, for the sake of clarity, in what follows the intense trailing sub-pulse will be referred to as the trailing pulse.

Although this scenario of nonlinear propagation applies to both diverging and converging pump beams, the differences in the generated SC spectra arise from slightly different spatio-temporal evolutions, which could be visualized through the differences in dynamics of the beam radii (Fig. 5a), achievable peak intensities (Fig. 5b) and peak plasma densities (Fig. 5c), which eventually are linked to the amounts of nonlinear losses. The diverging pump beam experiences less nonlinear absorption and creates a lower density free electron plasma, see the red curve in Fig. 5d. As a consequence, the leading pulse maintains high intensity for a longer propagation distance, see Fig. 5b, d, resulting in efficient red-shifted spectral broadening due to accumulation of the nonlinear phase (Fig. 5e). By contrast, the converging pump beam suffers much larger nonlinear losses as it self-focuses closer to the entrance face of the sample, producing a larger density of free electron plasma at the first nonlinear focus. As a result, the pump pulse experiences stronger defocusing of its trailing part, producing a leading pulse (which is responsible for the red-shifted spectral broadening) whose intensity drops faster with propagation, see Fig. 5b, f), resulting in just moderate spectral broadening towards the long-wavelength side (Fig. 5g). However, in this case, a more energetic trailing pulse is replenished, reaching a higher peak intensity at the secondary collapse point, thereby facilitating more efficient blue-shifted spectral broadening.

### Performance at 2 MHz pulse repetition rate

**Fig. 6:**
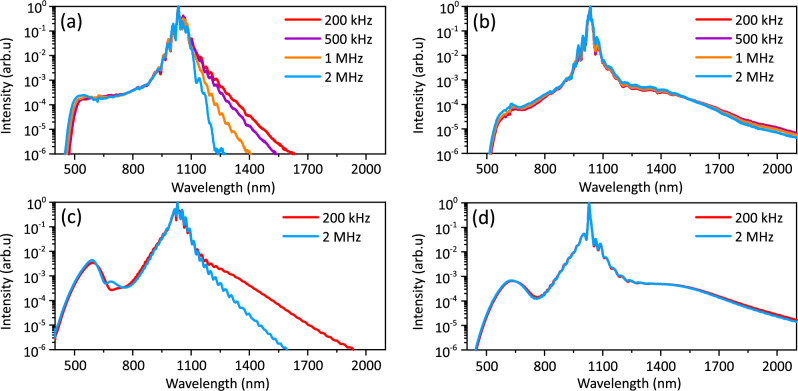
Supercontinuum spectra measured with (**a**) converging ($\Delta z=+1$ mm) and (**b**) diverging ($\Delta z=-1$ mm) pump beams at pulse repetition rates of 200 kHz, 500 kHz, 1 MHz and 2 MHz. (**c**,**d**) The respective numerically simulated supercontinuum spectra at 200 kHz and 2 MHz pulse repetition rates.

In what follows, we investigate the SC generation performances with converging ($\Delta z=+1$ mm) and diverging ($\Delta z=-1$ mm) pump beams that produce the largest spectral blue and red shifts, respectively, by increasing pulse repetition rate from 200 kHz to 2 MHz. The main experimental findings are illustrated in Fig. 6a, b, which present the SC spectra measured at pulse repetition rates of 200 kHz, 500 kHz, 1 MHz and 2 MHz, outlining remarkable differences in spectral behavior related to pump beam focusing geometry. A dramatic shrinking of the SC spectrum on the long-wavelength side with the increase of pulse repetition rate is recorded in the case of converging pump beam, with almost complete extinction of long-wavelength portion of SC spectrum at 2 MHz, as shown in Fig. 6a. The diverging pump beam produces SC spectrum of almost constant width regardless of pulse repetition rate, just showing a very minor reduction of spectral intensity of the most red-shifted components, see Fig. 6b. The experimental findings are reproduced by the numerical simulations shown in Fig. 6c, d, which compare the respective simulated SC spectra at pulse repetition rates of 200 kHz and 2 MHz.

The contrasted SC generation performances regarding pulse repetition rate and pump beam focusing could tentatively be attributed to heat accumulation. Comparison of the thermal diffusion length with the filament diameter may serve as a qualitative indicator of heat accumulation and the onset of thermal effects. The thermal diffusion length is defined as $L_\textrm{diff}=\sqrt{D/f}$, where *f* is the pulse repetition rate and $D=3.8$ mm$^2$/s is the thermal diffusivity of YAG at room temperature (300 K)^47^. At pulse repetition rate of 200 kHz, the calculated thermal diffusion length of $4.4~\upmu$m is close to filament diameter of $\sim 5 \, \upmu$m, which was estimated at the nonlinear focus, see Fig. 5a, so in this case, a large fraction of induced heat dissipates out of filament site before the next laser pulse arrives. This is not the case for the 2 MHz pulse repetition rate, where the calculated diffusion length of $1.4~\upmu$m is noticeably smaller than the filament diameter, suggesting efficient heat accumulation at the filament site with passage of every laser pulse. Next, since the locally induced temperature change is proportional to the amount of deposited energy (Eq. 11), we recall that under examined operating conditions there is a large difference of nonlinear losses experienced by converging and diverging pump beams. More specifically, 12% of the input energy is nonlinearly absorbed in the case of the diverging pump beam ($\Delta z=-1$ mm), while in the case of the converging pump beam($\Delta z=+1$ mm), the energy losses increase to 36%, as measured experimentally, with respective values of 8% and 28%, as evaluated from numerical simulations.

**Fig. 7:**
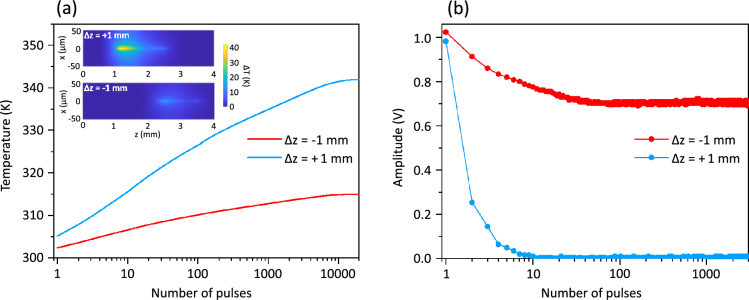
(**a**) The dynamics of accumulated temperature at the beam center induced by converging and diverging pump beams at 2 MHz pulse repetition rate. The insets show the respective temperature distributions at the filament site, see text for details. (**b**) Experimentally measured time evolutions of supercontinuum spectral amplitude in the 1500–1700 nm range.

Figure 7a presents the numerically simulated dynamics of the accumulated temperature at the beam center as a function of the number of pulses at 2 MHz pulse repetition rate in the cases of diverging and converging pump beams, assuming that the entire amount of deposited energy is converted into local heating of the material. The local temperature values are provided just before the arrival of the next laser pulse and were calculated by solving the thermal diffusion equation 13. In both cases the local temperature increases very rapidly with the first 10 laser pulses (note the logarithmic scale) and tends to settle after passage of $\sim 10000$ pulses. The insets show the respective temperature distributions at the filament site just before the arrival of the 10000th laser pulse. The converging pump beam induces a local temperature increase by $42^\circ$C at the beam center and the largest temperature gradient at the vicinity of the first nonlinear focus. Due to the positive thermo-optic coefficient of YAG, the induced thermal lens contributes to the linear and nonlinear focusing, altering propagation of the leading pulse in such a way that the red-shifted spectral broadening is suppressed by a faster decline of the leading pulse intensity. The diverging pump beam undergoes a lesser perturbation in its nonlinear propagation, as the local temperature increase does not exceed $15^\circ$C and the effect of thermal lensing is apparently weak.

The experimental and numerical data presented in Fig. 6 show the SC spectra at the time when the local temperature regime settles. The numerical data represent the simulated SC spectra generated by the 10000-th laser pulse, which “sees” local temperature distributions depicted in the insets of Fig. 7a, while the experimental spectra were measured after $\sim 5$ min of exposure time. However, the build-up of local heating is a dynamic process taking place on a characteristic time scale related to the time separation between adjacent laser pulses, as Fig. 7a suggests. To experimentally justify the relation between local temperature change and SC spectral red-shift, we measured the time evolution of the spectral amplitude of SC radiation in the 1500-1700 nm wavelength range. The results are illustrated in Fig. 7b which compares the experimentally measured pulse-to-pulse dynamics of the spectral amplitude in the selected spectral interval in the cases of diverging and converging pump beams. In the case of converging pump beam, the spectral amplitude drops to zero after just 10 laser pulses, indicating that the red-shifted portion of SC spectrum quickly shrinks beyond the detection window. The reduction of spectral amplitude was captured also in the case of diverging pump beam, however, the change of spectral amplitude does not exceed 30% (just about noticeable in the SC spectrum shown on a logarithmic intensity scale in Fig. 6b) and settles to a steady value after $\sim 100$ pulses, while the local temperature continues to slowly increase. The time evolution measurements performed at the 200 kHz pulse repetition rate (not presented here) revealed just a barely noticeable decrease (by less than 3%) of spectral amplitude in the case of diverging pump beam, suggesting that the role of thermal effects under these operating conditions could be largely neglected. A reduction in spectral amplitude of 35% was recorded with the converging pump beam and showed a very similar time evolution compared to that measured with the diverging pump beam at the pulse repetition rate of 2 MHz.

To summarize, the measured dynamics of SC signal amplitude in the 1500–1700 nm range at 2 MHz as well as at 200 kHz pulse repetition rates demonstrated that spectral changes occur on a time scale characteristic to build-up of local heating due to energy deposition via nonlinear absorption. However, the magnitude of induced spectral changes strongly depends not only on the pulse repetition rate but also on the amount of deposited energy, which is a function of the beam waist offset with respect to the input face of the sample and could be easily controlled experimentally. Finally, it should be noted that no permanent modification or damage of the nonlinear material was observed in any case of pump beam focusing, as verified by full recovery of SC spectral width while letting the crystal cool down and decreasing the pulse repetition rate from 2 MHz to 200 kHz.

## Conclusions

We performed a detailed experimental and numerical study of SC generation in a YAG crystal with 210 fs, 1030 nm pulses from an amplified Yb:KGW laser system, which uncovered relevant pump beam focusing geometry-related features of SC generation and underlying dynamics of nonlinear pulse propagation. It is demonstrated that the largest red shift of the SC spectrum is produced when entering the nonlinear material with a slightly diverging pump beam, whereas a slightly converging pump beam produces the SC spectrum with the largest spectral blue shift. These spectral features are related to different amounts of deposited energy via nonlinear losses experienced by converging and diverging pump beams, setting different thermal regimes of filamentation, which emerge by increasing the pulse repetition rate from 200 kHz to 2 MHz. In particular, it was found that the long-wavelength portion of SC spectrum generated with converging pump beam shrinks considerably with the increase of pulse repetition rate, and almost completely vanishes at 2 MHz. Such dramatic spectral shrinking is attributed to the build-up of thermal lens, which alters the dynamics of nonlinear propagation. These changes occur on a microsecond time-scale, as justified by numerically simulated dynamics of heat accumulation and experimentally measured pulse-to-pulse dynamics of SC spectral amplitude in the 1500-1700 nm wavelength range. On the other hand, owing to significantly reduced nonlinear losses and heat accumulation in the case of diverging pump beam, stable and broad supercontinuum spectrum is generated almost regardless of pulse repetition rate. These results are of importance for improving and optimizing high repetition rate SC generation stages used in high speed spectroscopic^48,49^ and imaging^50–52^ setups, where SC generation in bulk nonlinear crystals emerges as an appealing alternative to broadband sources based on photonic crystal fibers. More generally, these findings may extend to SC generation in normally dispersive nonlinear materials with a positive thermo-optic coefficient (dn/dT>0), and to laser pulsewidths producing similar temporal dynamics of filamentation and spectral broadening.

## Methods

### Numerical

The position of nonlinear focus for a collimated Gaussian beam could be accurately predicted by empirical Marburger‘s formula^53^:

1 $$\begin{aligned} z_\textrm{sf}=\frac{0.367z_R}{\sqrt{\left( \sqrt{\frac{P}{P_\textrm{cr}}}-0.852\right) ^2-0.219}}, \end{aligned}$$

where $z_R=\pi n_0 w^2/\lambda$ is the diffraction (Rayleigh) length of the beam with a waist radius *w*, $\lambda =1030$ nm is the laser wavelength, $n_0=1.8153$ is the linear index of refraction of YAG and $P=4.9$ MW is the peak power of the pump pulse with a duration of 210 fs and energy of $1.1~\mu$J (evaluated from the experimental value and accounting for Fresnel reflections from the lens and entrance face of the YAG crystal). $P_{cr}=3.77\lambda ^2/8\pi n_0 n_2$ is the critical power for self-focusing of a Gaussian beam, where $n_2$ is the nonlinear index of refraction. Considering variable position of the beam waist with respect to the entrance face of the sample, the beam diameter entering the sample was calculated as follows:

2 $$\begin{aligned} w=w_0\sqrt{1+\frac{\Delta z}{z_{R_0}}}, \end{aligned}$$

where $z_{R_0}=\pi w_{0}^2/\lambda$ is the Rayleigh length of the focused beam in air, $w_0=20~\mu$m is the beam waist radius at the $1/e^2$ intensity level and $\Delta z$ is the beam waist offset with respect to the entrance face of the crystal, as defined in the experiment, see the inset of Fig. 1. In this case, the curvature radius of the phase front in air was taken into account at the position of the sample entrance face:

3 $$\begin{aligned} R=\Delta z+z_{R_0}^2/\Delta z. \end{aligned}$$

The negative and positive *R* values correspond to diverging and converging input beams, respectively. Then, the modified position of the nonlinear focus is expressed as:

4 $$\begin{aligned} z_\textrm{sf}'=\frac{z_\textrm{sf}n_0R}{n_0R+z_\textrm{sf}}, \end{aligned}$$

This expression was used to fit the experimental data, taking the $n_2$ value as a free parameter. The best fit was obtained with the $n_2=5.2\times 10^{-16}$ cm$^2$/W, which was further used in the numerical simulations.

The nonlinear propagation of ultrashort pulses through the material was numerically simulated using a unidirectional nonparaxial nonlinear Schrodinger equation in the spectral domain^54^:

5 $$\begin{aligned} \frac{\partial S(\Omega ,k_{\perp })}{\partial z}+iD(\Omega ,k_{\perp })=S_N(\Omega ,k_{\perp }), \end{aligned}$$

where *S* is the pulse spectrum, terms *D* and $S_N$ stand for linear and nonlinear parts of the equation, respectively, $\Omega$ denotes the frequency shift from the carrier frequency $\omega _0$, $k_\perp$ is the transverse wave number, *z* is the propagation coordinate (propagation distance). The linear part of Eq. (5) is responsible for diffraction and dispersion, and is expressed in the form:

6 $$\begin{aligned} D(\Omega ,k_{\perp })=\sqrt{k(\omega _0+\Omega )^2-k_{\perp }^2}-k_0-\frac{\Omega }{v_g}, \end{aligned}$$

where $k(\omega )=n(\omega )\omega /c$ is the wavenumber, $n(\omega )$ is the refractive index of YAG taken from^55^, $v_g=\left.\frac{d\omega}{dk}\right|_{\omega=\omega_0}$ is the group velocity of the pulse, and $k_0=k(\omega _0)$ is the wavenumber at the carrier frequency. The nonlinear part of Eq. (5) is solved in the physical domain:

7 $$\begin{aligned} S_N(\Omega ,k_{\perp })=\int _{-\infty }^{+\infty }\int _{0}^{+\infty }N(t,r)e^{-i\Omega t}J_0(k_{\perp },r)rdrdt, \end{aligned}$$

where integration of $J_0$ Bessel zero order functions was used for inverse Hankel transform. The nonlinear term *N*(*t*, *r*) includes (from left to right) the instantaneous Kerr response, free-carrier generation via multiphoton absorption, plasma absorption and plasma-induced defocusing, and wavefront bending due to thermally induced refractive-index change:

8 $$\begin{aligned} N(t,r)=\frac{i\omega _0n_2n_0}{cn(w)}|A|^2A-\frac{\beta _{K}}{2}|A|^{2K-2}A-\frac{\sigma }{2}(1+i\omega _0\tau _c)\rho _e A+\frac{i\omega _0\Delta n(z,r)}{c}A, \end{aligned}$$

where *A* is the complex pulse amplitude, $\rho _e$ is the density of free electron plasma. *K* is the order of multiphoton absorption; in the present case $K=6$, assuming pump photon energy $\hbar \omega _0=1.2$ eV and YAG bandgap $U_g=6.5$ eV. The multiphoton absorption coefficient $\beta _K=6.9\times 10^{-64}cm^9/W^5$ was calculated from the Keldysh formalism. The plasma cross-section $\sigma$ was calculated using the following equation:

9 $$\begin{aligned} \sigma =\frac{e^2\tau _c}{cn_0\epsilon _0m(1+\omega ^2_0\tau ^2_c)}, \end{aligned}$$

where $\tau _c=1$ fs is the electron collision time, *e* and *m* are the electron charge and mass, respectively, *c* is the speed of light in a vacuum and $\epsilon _0$ is the vacuum permittivity, yielding $\sigma =1.3\times 10^{-21}$m$^2$. The time evolution of the electron plasma density is governed by:

10 $$\begin{aligned} \frac{\partial \rho _e}{\partial t}=\frac{\beta ^{K}}{K\hbar \omega_0 }|A|^{2K}+\frac{\sigma }{U_g}\rho _e A-\frac{\rho _e}{\tau _r}, \end{aligned}$$

where $\tau _r=150$ ps is the plasma recombination time in YAG^56^. The spatial distribution of the absorbed energy density due to multiphoton and free carrier absorption via inverse Bremsstrahlung effect, $u_\textrm{abs}(z,r)$, was calculated by integrating the real part of equation (8): $u_\textrm{abs}(z,r)=\int Re(-2NA^{*}) dt$.

In the thermal model, all absorbed energy is assumed to be converted into lattice heating:

11 $$\begin{aligned} u_\textrm{abs}=\int _{T_{n-1}}^{T_{n-1}+\Delta T}C_p(T')\rho _m(T')dT', \end{aligned}$$

where $C_p$ is the specific heat and $\rho _m$ is the density of the material. $T_{n-1}$ denotes the local temperature before the arrival of the *n*-th laser pulse, and $T_n=T_{n-1}+\Delta T$ is the resulting temperature after its passage. Minor energy loss channels (e.g. radiative plasma recombination, carrier transport, trapping, and pressure wave generation) were neglected, and this assumption may slightly overestimate the temperature rise. The heat diffusion was simulated by solving the thermal diffusion equation:

12 $$\begin{aligned} \rho _m(T) C_p(T) \frac{\partial t}{\partial T}=\nabla \cdot (\kappa (T) \nabla T) \end{aligned}$$

where $\kappa$ is the thermal conductivity. The temperature distribution was converted into the refractive index change:

13 $$\begin{aligned} \Delta n(z,r)=T(z,r) \frac{dn}{dT} \end{aligned}$$

noted in Eq. 8, where *dn*/*dT* is the thermo-optic coefficient. The calculations were performed with relevant values of $C_p=0.604$ J/gK, $\rho _m=4.552$ g/cm$^3$, $\kappa =10.4$ W/mK and $dn/dT=7.5\times 10^{-6}$ K$^{-1}$ of the YAG crystal at room temperature (300 K) and taking into account their temperature dependencies^47^. The complex pulse amplitude at the input ($z=0$) was defined as:

14 $$\begin{aligned} A(r,t)=A_0exp\left( -2ln2\frac{t^2}{\tau _{FWHM}^2}-\frac{r^2}{w^2}-i\frac{k_0}{2}\frac{r^2}{n_0R}\right) \end{aligned}$$

where $A_0$ is the peak amplitude, *w* and *R* are the input beam radius and wavefront curvature computed using Eqs. (2) and (3), respectively, while $\tau _{FWHM}=210$ fs is the pulse duration defined at full width at half maximum.

To reduce computational time for numerical simulations, we implemented the following simulation strategy. Since the most significant temperature changes occur with propagation of the very first pulses and progressively saturate as the number of pulses increases, it is not necessary to compute the full nonlinear propagation for every individual pulse. At larger pulse numbers, the pulse-to-pulse variation in the heat distribution becomes negligible. This observation enables to reduce time-consuming pulse-propagation calculations by assuming a constant deposited energy density distribution over a defined number of consecutive pulses, while heat diffusion is performed for each pulse. In doing so, the pulse sequence was sampled using propagation of 1, 2, 5, 10, 100, etc. pulses.

## Data Availability

The datasets used and/or analysed during the current study available from the corresponding author on reasonable request.
